# Genetic epilepsies with myoclonic seizures: Mechanisms and syndromes

**DOI:** 10.1002/epi4.70039

**Published:** 2025-04-09

**Authors:** Antonietta Coppola, Marica Rubino, Antonella Riva, Pasquale Striano

**Affiliations:** ^1^ Department of Neuroscience, Reproductive and Odontostomatological Sciences Federico II University Naples Italy; ^2^ Neurology Unit University Hospital Federico II Naples Italy; ^3^ Department of Neurosciences, Rehabilitation, Ophthalmology, Genetics, Maternal and Child Health University of Genoa Genoa Italy; ^4^ IRCCS G. Gaslini Full Member of Epicare Genoa Italy

**Keywords:** epilepsy, genetics, myoclonic, non‐coding expansions

## Abstract

Genetic epilepsy with myoclonic seizures encompasses a heterogeneous spectrum of conditions, ranging from benign and self‐limiting forms to severe, progressive disorders. While their causes are diverse, a significant proportion stems from genetic abnormalities. Advances in genetic research over the past decade have uncovered novel mechanisms underlying these disorders, including single‐gene mutations, copy number variations (CNVs), and non‐coding repeat expansions. These findings not only deepen our understanding of their pathophysiology but also highlight potential avenues for precision medicine. This review provides a comprehensive overview of myoclonic epilepsies linked to pathogenic gene variants, exploring genetic mechanisms and discussing their clinical implications.

**Plain Language Summary:**

In this work, we describe genetic epilepsies mainly characterized by myoclonic seizures, their genetic defects and disease mechanisms, and considerations of precision medicine treatment.

**Key points**

- Myoclonic epilepsies are often caused by a genetic etiology, including single‐gene mutations, copy number variations, and non‐coding repeat expansions.
- The discovery of a genetic etiology has prognosis, counseling, and therapeutic implications.
- Precision medicine treatment is available only for some cases but is destined to have larger applications in the future.

## 1 INTRODUCTION

Myoclonic epilepsies (MEs) represent a large and heterogeneous group of conditions. The disease course can be very different, ranging from benign to severe and progressive syndromes. Myoclonic seizures may present as one of the different seizure types experienced by the patient or be the only seizure type; they may appear or disappear at certain ages; they can be the only neurological phenomenon or be accompanied by other neurological manifestations.^1^ Although the causes can be heterogeneous as well, a significant proportion is caused by a genetic defect. Identifying the underlying genetic etiology is very important for treatment, prognosis, and counseling implications. Indeed, beyond the importance of avoiding certain medications that may aggravate the disorder, new therapeutic agents based on precision medicine treatment are currently being studied for some of these genetically determined MEs.

The advances in genetic technology of recent years, including exome sequencing, have exponentially increased the numbers of genes associated with MEs. In addition, genome‐wide technologies have disclosed new mechanisms intervening in the pathophysiology of certain MEs, going beyond mendelian and mitochondrial inheritance, such as non‐coding repeat expansions.^2^

We aim at narratively reviewing the genetic form of MEs covering the known associated genes and genetic mechanisms underlying these conditions, including single‐gene mutations, CNVs, and non‐coding repeat expansions (summarized in Table 1).

**TABLE 1:** Overview of known myoclonic epilepsy syndromes, with causal genes and patterns of inheritance (defined or postulated).

Syndrome	Gene	Chromosomal location	Inheritance	OMIM	References	
**Myoclonic epilepsy associated with single‐gene mutation**						
EIDEE	*CDKL5* *KCNB1* *STXBP1* *SCN8A* *RARS2* *SCN1A* *SIK1* *TBC1D24*	Xp22.13 20q13.13 9q34.11 12q13.13 6q15 2q24.3 21q22.3 16p13.3	X‐linked dominant Autosomal dominant Autosomal dominant Autosomal dominant Autosomal recessive Autosomal dominant Autosomal dominant Autosomal recessive	300 203 600 397 602 926 600 702 611 524 619 317 605 705 613 577	^3^ ^4^ ^5^ ^6^ ^7^ ^8^ ^9^ 10, 11	
Dravet syndrome	*SCN1A* *KCNQ2* *GABRA1* *STXBP1* *NEXMIF* *SLC2A1* *CDKL5*	2q24.3 20q13.33 5q34 9q34.11 Xq13.3 1p34.2 Xp22.13	Autosomal dominant Autosomal dominant Autosomal dominant Autosomal dominant X‐linked dominant Autosomal dominant X‐linked dominant	607 208	^12^ 13, 14 13, 14 13, 14 13, 14 13, 14 13, 14	
EEM EEM+	*GABRA1* *RORB* *CHD2* *SYNGAP1* *TRIM8* *NEXMIF*	5q34 9q21.13 15q26.1 6p21.32 10q24.32 Xq13.3	Autosomal dominant Autosomal dominant Autosomal dominant Autosomal dominant Autosomal dominant X‐linked dominant	611 136 618 357 621 012 612 621 300 912	^15^ ^16^ ^17^ ^18^ ^17^ ^17^	
RETT syndrome	*MECP2*	Xq28	X‐linked dominant	3 112 750	^19^	
Angelman syndrome	*UBE3A* 15q11.3‐q13 deletion uniparental disomy (imprinting defect)	15q11.2	Autosomal dominant (Loss of function of the maternal copy)	105 830 105 830	20, 21 20, 21	
Huntington's disease	*HTT*	4p16.3	CAG triplets (autosomal dominant)	613 004	^22^	
**Progressive myoclonic epilepsies (PMEs)**						
Unverricht‐Lundborg disease (EPM1)	CSTB	21q22.3	Autosomal recessive (dodecamer expansion or point mutation)	254 800	^23^	
*PRICKLE1*‐related PME	*PRICKLE1*	12q12	Autosomal recessive	612 437	^24^	
*SCARB2*‐ralated PME	*SCARB2*	4q21.1	Autosomal recessive	254 900	^25^	
Sialidosis type I	*NEU1*	6p21.33	Autosomal recessive	256 550	^26^	
*KCNC1*‐related PME (or MEAK)	*KCNC1*	11p15.1	Autosomal dominant	616 187	^27^	
*GOSR2*‐related PME	*GOSR2*	17q21.32	Autosomal recessive	614 018	^27^	
*ATP6VOA1‐related* PME	*ATP6VoA1*	17q21.2	Autosomal recessive		^28^	
Lafora disease (EPM2A/B)	*EPM2A* *NHLRC1/EPM2B*	6q24.3 6q22.3	Autosomal recessive	254 780 620 681	29, 30 29, 30	
Ceroid lipofuscinosis type 2	*TPP1*	11p15.4	Autosomal recessive	204 500	31, 32	
Ceroid lipofuscinosis type 3	*CLN3*	16p12.1	Autosomal recessive	204 200	31, 32	
**Progressive myoclonic epilepsies (PMEs)**						
Ceroid lipofuscinosis type 4	*DNAJC5*	20q13	Autosomal recessive o dominant		162 350	31, 32
Ceroid lipofuscinosis type 5	*CLN5*	13q22.3	Autosomal recessive		256 731	31, 32
Ceroid lipofuscinosis type 6	*CLN6*	15q23	Autosomal recessive		601 780	31, 32
Ceroid lipofuscinosis type 7	*MFSD8*	4q28.2	Autosomal recessive		610 951	31, 32
Ceroid lipofuscinosis type 8	*CLN8*	8p23.3	Autosomal recessive		600 143	31, 32
Ceroid lipofuscinosis type 10	*CTSD*	11p15.5	Autosomal recessive		610 127	31, 32
Ceroid lipofuscinosis type 11	*GRN*	17q21.31	Autosomal recessive		614 706	31, 32
Ceroid lipofuscinosis type 12	*ATP13A2*	1p36.13	Autosomal recessive		606 693	31, 32
Ceroid lipofuscinosis type 13	*CTSF*	11q13.2	Autosomal recessive		615 362	31, 32
Ceroid lipofuscinosis type 14	*KCTD7*	7q11.21	Autosomal recessive		611 726	31, 32
SMA‐PME	*ASAH1*	8p22	Autosomal recessive		159 950	^33^
*CERS1*‐related PME	*CERS1*	19p13.11	Autosomal recessive		616 230	^34^
*GBA*‐related PME	*GBA*	1q22	Autosomal recessive			^34^
*SACS*‐related PME	*SACS*	13q12.12	Autosomal recessive			^34^
*CACNA2D2*‐related PME	*CACNA2D2*	3p21.31	Autosomal recessive			^34^
*STUB1*‐related PME	*STUB1*	16p13.3	Autosomal recessive			^34^
*AFG3L2*‐related PME	*AFG3L2*	18p11.21	Autosomal recessive			^34^
*NAXE*‐related PME	*NAXE*	1q22	Autosomal recessive		618 876	^34^
*ALG10*‐related PME	*ALG10*	12p11.1	Autosomal recessive			^34^
*CHD*2‐related PME	*CHD2*	15q26.1	Autosomal dominant			17, 28
*NUS1*‐related PME	*NUS1*	6q22.1	Autosomal dominant			17, 28
*SEMA6B*‐related PME	*SEMA6B*	19p13.3	Autosomal dominant		618 866	17, 28
*DHDDS*‐related PME	*DHDDS*	1p36.11	Autosomal dominant		617 836	17, 28
*DYNC1H1*‐related PME	*DYNC1H1*	14q32.31	Autosomal dominant			^35^
**Mitochondrial myoclonic epilepsy**						
MERFF	*MTTK*	Mitochondrial DNA at position 12	Maternal line	545 000	^36^	
*POLG*‐related syndrome	*POLG1*	15q26.1	Autosomal recessive	607 459 203 700	^36^	
**Myoclonic epilepsy associate with CNVs**						
Down syndrome	21 trisomy		Autosomal dominant	190 685	^37^	
Pallister‐Killian syndrome	Mosaic 12p tetrasomy			601 803	^38^	
**Myoclonic epilepsy associated with non‐coding variants**						
*Familial adult myoclonus epilepsy*						
FAME1	*SAMD12*	8q24.11‐q24.12	Autosomal dominant	601 068	^39^	
FAME2	*STARD7*	2q11.2	Autosomal dominant	607 876	^40^	
FAME3	*MARCH6*	5p15.2	Autosomal dominant	613 608	^41^	
FAME4	*YEATS2*	3q27.1	Autosomal dominant	615 127	^42^	
FAME6	*TNRC6a*	16p12.1	Autosomal dominant	618 074	^43^	
FAME7	*RAPGEF2*	4q32.1	Autosomal dominant	618 075	^39^	
FAME8	*RAI1*	17p11.2	Autosomal dominant		^43^	

Abbreviations: EEM, epilepsy with eyelid myoclonia; EIDEE, early infantile developmental and epileptic encephalopathy; FAME, familial adult myoclonic epilepsy; MERFF, mitochondrial encephalomyopathy with ragged‐red fibers; mtDNA, mitochondrial DNA; PME, progressive myoclonus epilepsies; POLG, mitochondrial DNA polymerase gamma; SMA‐PME, childhood spinal muscular atrophy (SMA) associated with PME.

## 2 MYOCLONIC EPILEPSY ASSOCIATED WITH SINGLE‐GENE MUTATION

### 2.1 Early infantile developmental and epileptic encephalopathy (EIDEE)

EIDEE are severe developmental and epileptic encephalopathies (DEEs) of early infancy, characterized by an onset of seizures within the first 3 months of life; seizures are commonly frequent and drug‐resistant. Interictal EEG shows abnormal background activity typically with burst‐suppression, multifocal spikes/spike waves/sharp waves with or without slowing, discontinuity and/or diffuse slowing. Abnormal neurological signs and developmental impairment are a constant finding as they usually manifest even before seizure onset. Although infants with EIDEE may experience different types of seizures and causative pathogenic gene variants can be identified in more than half of patients, myoclonic seizures can be the hallmark of some of them.^13^

The EIDEE mainly featuring myoclonic seizures is associated with pathogenic variants in the following genes: *CDKL5*, X‐linked dominant (OMIM #300203)^3^; *KCNB1*, autosomal dominant (OMIM #600397) *[5]; STXBP1*, autosomal dominant (OMIM #602926)^5^ *; SCN8a* autosomal dominant (OMIM #600702)^6^; *RARS2*, autosomal recessive (OMIM #611524)^7^; *SCN1A*, autosomal dominant (OMIM #619317)^8^; *SIK1*, autosomal dominant (OMIM #605705)^9^; *TBC1D24*, autosomal recessive (OMIM #613577).^10^, ^11^

### 2.2 Dravet syndrome (DS)

Dravet syndrome represents one of the most studied and known DEE. Myoclonic seizures are very typical of DS, although they are not the type of seizure at onset. The onset is typically characterized by prolonged febrile convulsions within the first year of life. Temperature sensitivity lasts until late adolescence–adulthood. Seizures are classically resistant to antiseizure medications (ASM) and are accompanied from the second year of life by cognitive and behavioral impairments. Subsequently, children also develop other neurological symptoms such as ataxia and a characteristic gait disturbance known as crouch gait.

The clinical diagnosis is supported by the identification of pathogenic variants in the subunit of the sodium channel gene *SCN1A* (found in nearly 90% of individuals) including missense mutation, nonsense, frameshift, splice site, and a minority (10%) of in‐frame insertion/deletion and CNVs (OMIM #607208).^12^ More than 95% of these mutations arise de novo. There is some genotype–phenotype correlation, as truncating mutations are associated with earlier onset of seizures and a more severe phenotype.^12^ The hypothesis underlying DS is that the impaired function of *SCN1A* affects specifically inhibitory interneurons.

In the remaining cases, pathogenic variants affecting different genes, for example, *KCNQ2*, *CDKL5*, *GABRA1, STXBP1, NEXMIF*, and *SLC2A1* can be identified.^13^, ^14^ The clinical course and developmental impairment can show a certain degree of phenotypic variability depending on the gene‐specific syndrome.^13^

### 2.3 Epilepsy with eyelid myoclonia

**FIGURE 1:**
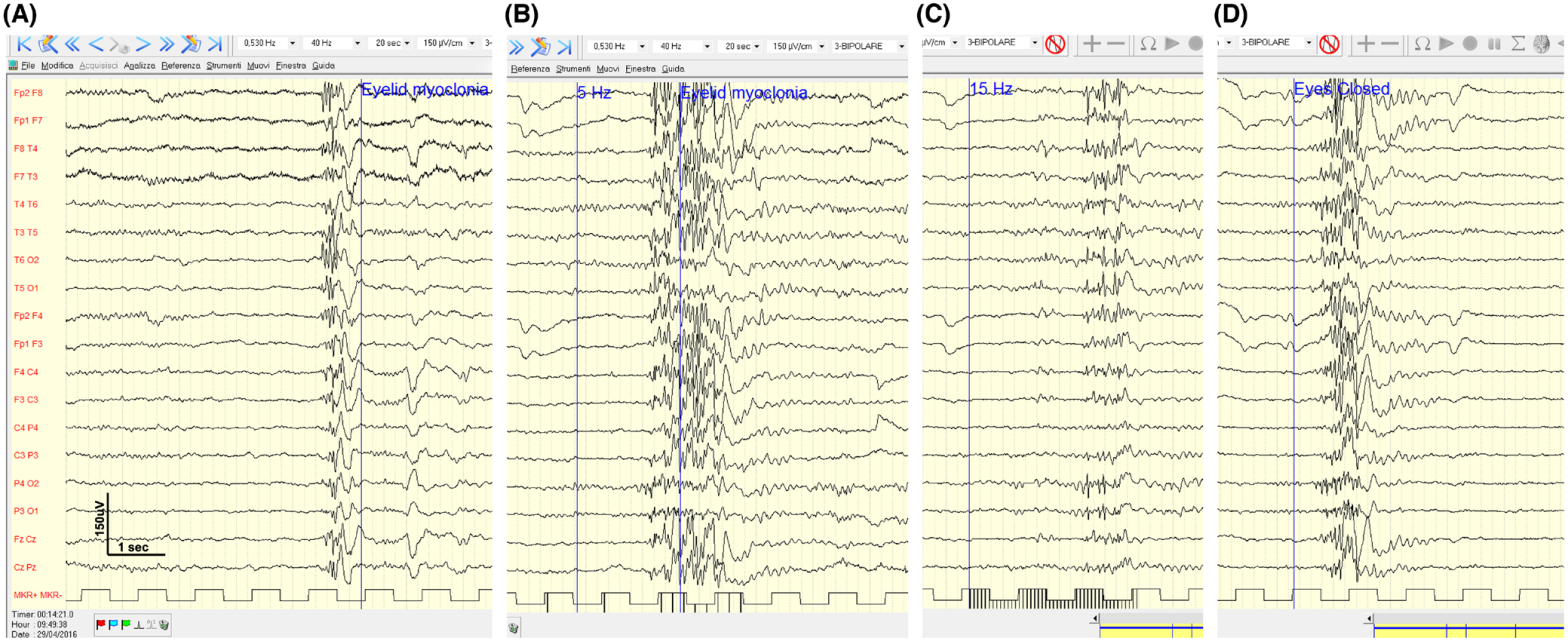
Electroencephalogram of an 8‐year‐old patient with epilepsy with eyelid myoclonia. (A) Spontaneous generalized polyspikes‐and‐wave discharge lasting less than a second, clinically associated with eyelid myoclonia. (B) Generalized polyspikes‐and‐wave discharge lasting 2 s is recorded during intermittent photic stimulation (5 Hz), clinically associated with eyelid myoclonia. (C) Interictal generalized polyspikes‐and‐wave discharge recorded during intermittent photic stimulation (15 Hz) lasting 1 s. (D) Generalized polyspikes‐and‐wave discharge lasting 2 s at eye closure clinically associated with eyelid myoclonia.

Epilepsy with eyelid myoclonia (EEM) is a generalized form of epilepsy clinically characterized by eyelid myoclonia with or without absences, eye closure‐induced seizures with EEG paroxysms, and photosensitivity^44^, ^45^ (Figure 1).

Clinical observations and recent genetic studies suggest that EEM is a spectrum of conditions, where EEM with comorbidities may be a different entity from isolated EEM. When EEM is associated with intellectual disability and/or behavioral comorbidities (EEM+), it is more likely to find a genetic cause by exome sequencing. Indeed, pathogenic variants affecting *CHD2* (OMIM #621012), *NEXMIF1* (OMIM #300912), and *TRIM8* are found in nearly 30% of EEM+ individuals. Among these pathogenic variants, *CHD2* and EEM+ seem to be the strongest association.^17^ Pathogenic variants in *GABRA1* (OMIM #61136) and *RORB* (OMIM #618357) have been reported in individuals with EEM^15^, ^16^; however, almost the totality of individuals with pure EEM remains negative at clinically available genetic testing.^17^ If a common variant exists for EEM, this remains to be elucidated, and it may emerge from studies of common variation, lie in oligo‐ or polygenic frameworks, or non‐coding sequences.

**FIGURE 2:**
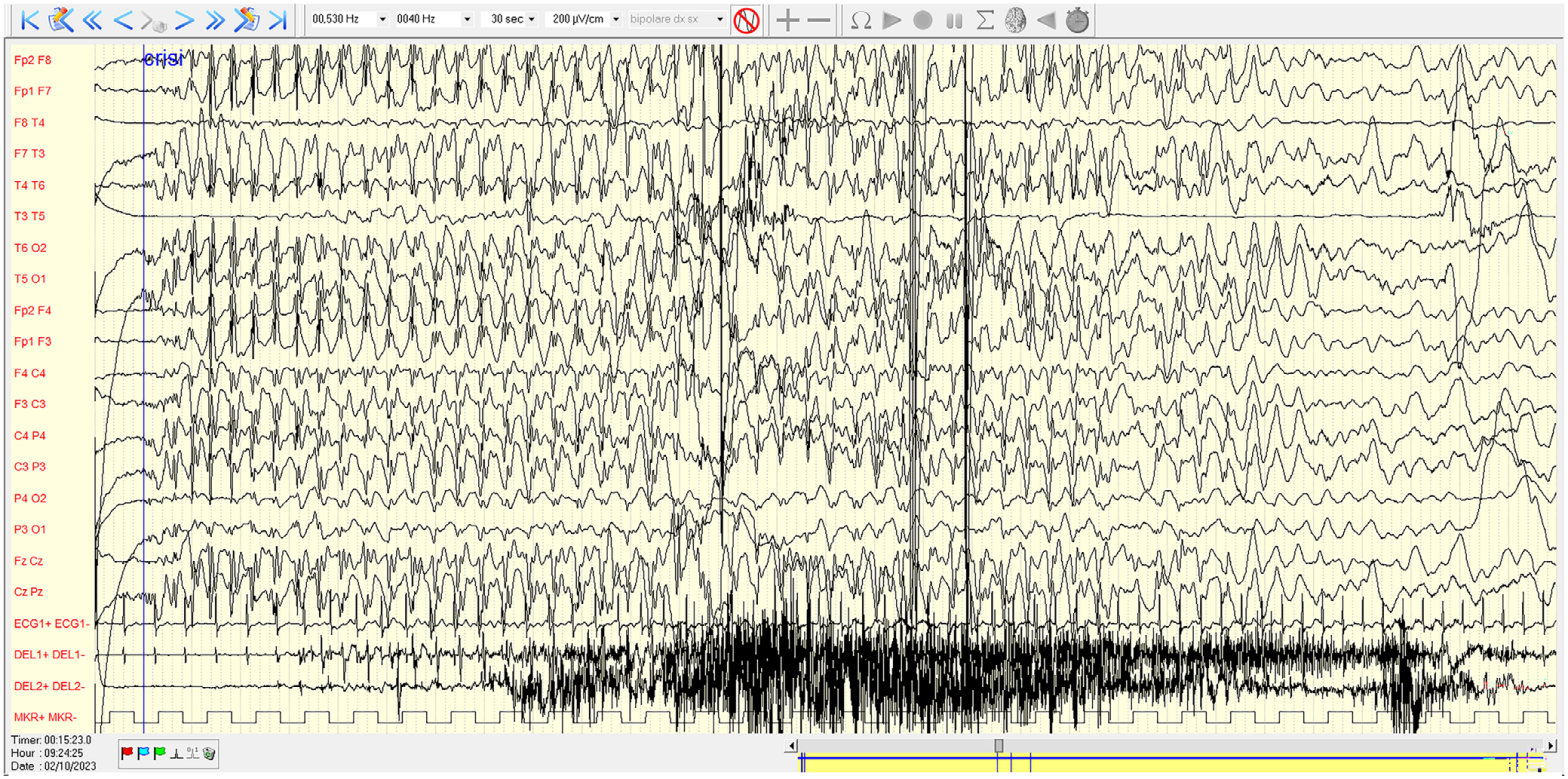
Ictal polygraphic (EEG‐EKG‐EMG: DEL1 left deltoid, DEL2 right deltoid) recording of a myoclonic absence in a 6‐year‐old child with DEE related to a *SYNGAP1* de novo mutation. Ictal discharge is characterized by a paroxysmal generalized 2‐Hz spike or polyspikes‐and‐wave complexes discharge. Clinically, the patient shows impaired awareness with head and arms myoclonias. Three seconds after the onset of the EEG discharge, bilateral synchronous myoclonic jerks timed with the polyspikes‐and‐wave complexes, subsequently partially masked by muscle artifact, are seen on electromyographic channels.

Rare variants affecting *SYNGAP1* can also be associated with an EEM+ phenotype and an epilepsy with myoclonic and atonic seizures phenotype (Figure 2). However, this condition is usually more complex and is often counted as a DEE in its own right. Indeed, rare variants in *SYNGAP1* cause a generalized DEE with a distinctive syndrome combining epilepsy with eyelid myoclonia with absences and myoclonic–atonic seizures (OMIM #612621). These patients can often suffer from reflex seizures triggered by eating, sound, or touch. Typically, seizures occur in clusters.^18^

### 2.4 Rett syndrome

Patients with classic Rett syndrome due to *MECP2* mutation (OMIM #3112750) may show a distinctive pattern of cortical reflex myoclonus, clinically characterized by multifocal, arrhythmic, and asynchronous jerks mainly involving distal limbs.^19^ Neurophysiology investigations can help differentiate this myoclonic pattern from other movement disorders in Rett syndrome. Indeed, jerk‐locked back averaging‐EEG (JL‐back AVG‐EEG) generating a contralateral centroparietal pre‐myoclonus transient wave preceding the jerk, enlarged somatosensory evoked potentials, and prolonged C‐reflex can confirm the clinical suspicion.^19^

### 2.5 Angelman syndrome (AS)

Myoclonic seizures are common in AS, typically occurring in young children and associated with spike‐and‐wave discharges on EEG. Myoclonic status is often associated with developmental regression. Indeed, seizures are always accompanied by severe intellectual disability, ataxia, tremulousness, and jerky non‐epileptic movements. This non‐epileptic myoclonus has no EEG correlation, develops in adolescence or early adulthood, and has no alteration with consciousness.^46^

AS is due to lack of genetic contribution from maternal chromosome 15q11‐13. This region encompasses three GABAA receptor subunit genes (beta3, alpha5, and gamma3).^20^ Analysis of parent‐specific DNA methylation imprints in the 15q11.2‐q13 chromosome region detects approximately 80% of individuals with AS, including those with a deletion, uniparental disomy, or an imprinting defect; fewer than 1% of individuals have a cytogenetically visible chromosome rearrangement (e.g., translocation or inversion). *UBE3A* sequence analysis detects pathogenic variants in an additional approximately 11% of individuals (OMIM 105830).^21^

### 2.6 Huntington's disease (HD)

Myoclonus might be encountered among the symptoms of Juvenile HD (onset before the age of 20) in combination with extrapyramidal symptoms, behavioral issues, and other seizure types. Myoclonus may be either generalized or associated with spike‐and‐wave complexes or multifocal, with features compatible with cortical reflex myoclonus.^22^

HD is due to the abnormal multiplication of CAG triplets (more than 36–39) in the *HTT* gene located on 4p16.3 (OMIM #613004). Early onset of the disorder correlates with very long CAG repeats. A severe phenotype consistent with PME has been described in children with CAG repeat expansion of more than 100 repeats. In these children, rigidity may be replaced by hypotonia.^22^, ^44^

### 2.7 Progressive myoclonus epilepsies

Progressive myoclonus epilepsies (PMEs) comprises a group of rare progressive disorders transmitted in most cases in an autosomal recessive manner. Myoclonus is the hallmark of these conditions that can manifest spontaneously or be provoked by action and sensitive stimuli such as light, touch, and sound. Besides myoclonus, patients experience epileptic seizures, cognitive decline, and cerebellar involvement manifesting with ataxia and dysarthria. Symptoms are inexorably progressive.

**FIGURE 3:**
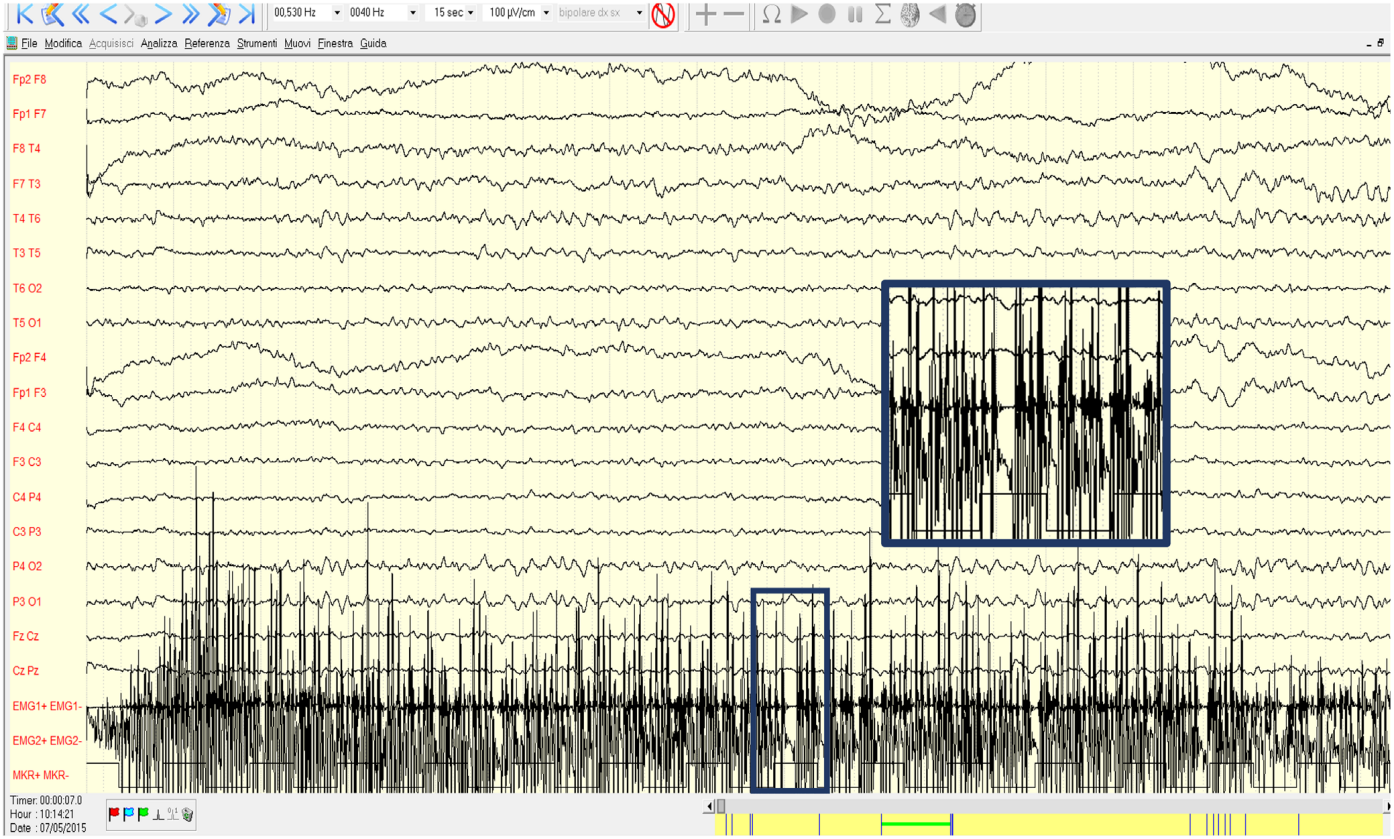
Polygraphic (EEG‐ECG‐EMG) recording in a patient with Unverricht‐Lundborg disease (EPM1) due to a homozygous unstable expansion of a 12‐nucleotide dodecamer repeat (5′‐CCCCGCCCCGCG‐ 3′) in *CSTB*. The image shows a slow and dysregulated background activity (6 Hz). Electromyographic channels (EMG1: Left deltoid, EMG2: Right deltoid) while patient is holding Mingazzini I position show a sustained increase in muscle tone with interspersed short traits of suppression of muscular electrical activity (negative myoclonus) in the absence of a simultaneous electroencephalographic discharge (black box).

Progressive myoclonus epilepsie can be summarily grouped into PME without early cognitive decline and PME with early cognitive decline. Among the first group, Unverricht‐Lundborg disease, also known as EPM1, represents the most frequent, accounting for more than one‐third of the patients.^29^ The onset is during adolescence with tonic–clonic seizures and reflex myoclonus. Ataxia, dysarthria, and incoordination develop later. Cognitive abilities remain normal for years until the slow decline with time is seen. EPM1 is caused by an unstable dodecamer (12 bp) unit of 5′‐ccccgccccgcg‐3′ located at the promoter region of the Cystatin B (*CSTB*) gene in the vast majority (92%) of patients (OMIM #254800) (Figure 3). The remaining patients have a biallelic single nucleotide variant and show a less severe phenotype^23^ Unlike trinucleotide repeats, known to have a high degree of intergenerational instability, the EPM1‐associated minisatellite does not appear to be as unstable, and anticipation is not known to occur.^23^

Another PME clinically resembling EPM1, although with prominent ataxia, can be bound in association with biallelic mutations affecting *PRICKLE1* (OMIM #612437).^24^ A further one similar to EPM1 in patients with renal failure preceding or following the onset of myoclonus is associated with biallelic mutations affecting *SCARB2* (OMIM #254900).^25^

Sialidosis type 1 caused by homozygous mutations affecting Neuraminidase 1 (NEU1) manifests with prominent cerebellar involvement, action myoclonus, cherry red spot in the macula, and progressive vision loss (OMIM #256550).^26^

A homozygous mutation in the Golgi SNAP receptor complex 2 gene (*GOSR2*) (OMIM #614018) can also provoke a PME with early‐onset ataxia (average 2 years of age), followed by myoclonic seizures after a few years. Patients can develop multiple and photosensitive seizures and scoliosis by adolescence, which represents a distinctive feature. Other skeletal abnormalities, including pes cavus and syndactyly, can occur. Patients become wheelchair bound in about 10 years from onset.^27^

One of the few autosomal dominant PMEs is now known as “MEAK” (Myoclonus Epilepsy and Ataxia due to Potassium channel mutation OMIM #616187), as this is caused by a heterozygous rare variant affecting *KCNC1* encoding KV3.1, a subunit of the KV3 voltage‐gated K+ channels, responsible for high‐frequency neuronal firing. MEAK and EPM1 have overlapping ages of onset and moderate to severe incapacitating myoclonus, infrequent tonic–clonic seizures, and mild, if any, cognitive decline. Differences emerge as the clinical course for MEAK is generally more severe.^47^

Biallelic variants in *ATP6V0A1* present with a phenotype of early‐onset PME with ataxia, while individuals carrying de novo missense variants show severe developmental and epileptic encephalopathy.^28^

**FIGURE 4:**
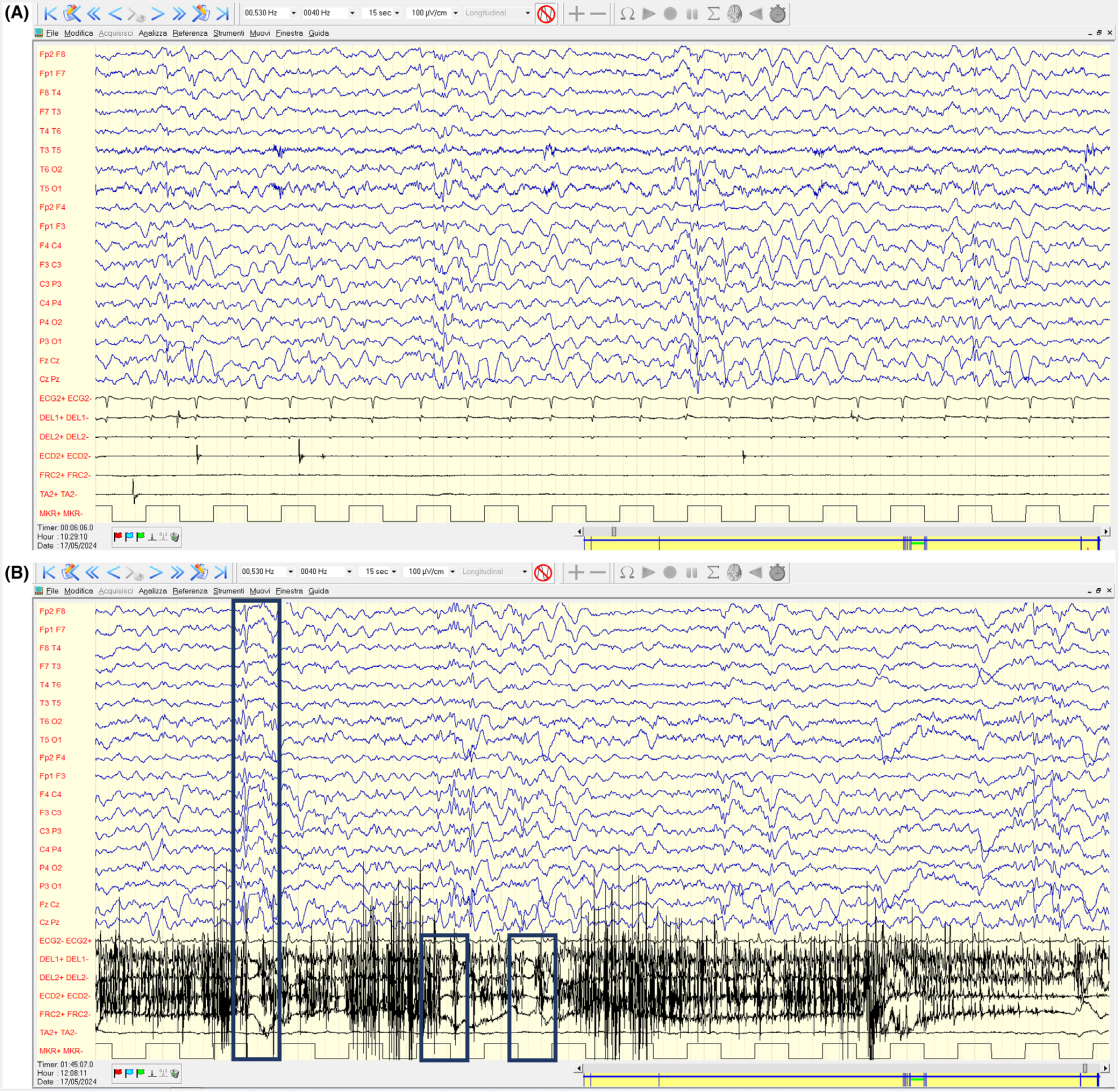
Polygraphic (EEG‐ECG‐EMG: DEL2: Right deltoids, DEL1: Left deltoids, ECD2: Right common extensor digitorum, FRC2: Right flexor carpi radialis), recording in a patient with Lafora disease due to a EPM2A homozygous mutation (A) The image shows a slow and disorganized background activity (4 Hz), frequent bilateral fronto‐central delta activity and bilateral spike/polyspikes‐wave discharges. On electromyogram asynchronous and arrhythmic bursts (<50 ms) of myoclonic activity with or without a simultaneous electroencephalographic discharge are recorded. (B) Electromyographic channels, while patient holds Mingazzini I position, show a sustained increase in muscle tone interrupted by traits of suppression of muscular electrical activity (negative myoclonus) in all muscles explored, with a simultaneous electroencephalographic discharge (Black Box).

Among the group with early cognitive decline, Lafora disease, also known as EPM2, is the most common, which is also the second most frequently occurring form of PME overall. EPM2 is an autosomal recessive PME with onset in early adolescence in otherwise neurologically normal individuals. Onset is typically characterized by myoclonic, tonic–clonic, or focal (occipital) seizures. Photosensitivity is very common and pronounced at onset. Ataxia and dementia emerge within 1 year from onset. Spontaneous and action myoclonus, both positive and negative, rapidly become severe, further challenging autonomous walking (See Figure 4A,B). Life expectancy is commonly 12 years; death is usually due to respiratory failure.^30^ In about 70% of patients, a biallelic mutation affecting *EPM2a* (OMIM #254780) coding for laforin can be detected. The remaining cases have biallelic mutations affecting EPM2b, also known as *NHLRC1*, coding for malin (OMIM #620681). Patients with EPM2b have a milder course.^29^, ^30^

Although the exact function of laforin and malin is not fully elucidated, the pathogenesis of EPM2 is known to be caused by the accumulation of polyglucosans within the brain, with subsequent neurodegeneration.

Neuronal ceroid lipofuscinoses (NCL) are a heterogenous and very rare group of inherited lysosomal storage disorders featuring PME commonly associated with early impaired intellectual abilities and visual impairment. The polymorphic clinical pictures include childhood onset with polymorphic seizures, intellectual disability, and late cortical myoclonus leading to premature death to a severe and almost pure myoclonic phenotype in adults. More than a dozen genes underlying human NCLs have been identified. However, the function of their protein products is not known in all subtypes. These genes encode lysosomal enzymes (CLN1 */PPT1* OMIM #256730, CLN2*/TPP1* OMIM #204500, CLN10/*CTSD* OMIM #610127, CLN13*/CTSF* OMIM #615362), a soluble lysosomal protein (CLN5) (OMIM #256731), a protein in the secretory pathway (CLN11/*GRN*) (OMIM #614706), two cytoplasmic proteins that also peripherally associate with membranes (CLN4/*DNAJC5* OMIM #162350, CLN14/*KCTD7* OMIM #611726), and many transmembrane proteins with different subcellular locations (CLN3 OMIM #204200, CLN6 OMIM #601780, CLN7/*MFSD8* OMIM #610951, CLN8 OMIM #600143, CLN12/ *ATP13A2* OMIM #606693). Mutated CLN4/*DNAJC5* is responsible for the rare dominant adult type (CLN4).^31^, ^32^ The presence of strong and early photosensitivity at low stimulation frequency (1–3 Hz) is considered a hallmark of CLN2 disease.^48^

Childhood spinal muscular atrophy (SMA) associated with PME (SMA‐PME) has been reported as a rare autosomal recessive condition linked to mutations in the N‐acylsphingosine amidohydrolase 1 (*ASAH1*) gene (OMIM #159950). The onset is in childhood with both features of SMA (proximal muscular weakness) and generalized epilepsy (absences and myoclonic seizures). Cognition is soon impaired, although at various degrees. Neurophysiological evaluation discloses both positive and negative myoclonus. Abnormal auditory and visual evoked potentials are also a feature.^33^

Likely pathogenic variant associated with a PME phenotype has been reported in anecdotal cases in other genes including *CERS1* OMIM #616230, *GBA, SACS, CACNA2D2, STUB1, AFG3L2, and NAXE* OMIM #617186, *ALG10*, associated with an autosomal recessive inheritance.^34^ Heterozygous variants have been reported in *CHD2, NUS1* OMIM #617831, *SEMA6B* OMIM #618876, *and DHDDS* OMIM #617836, *DYNC1H1*.^17^, ^28^, ^34^, ^35^

## 3 MITOCHONDRIAL MYOCLONIC EPILEPSY

Genetic disorders affecting mitochondria include those due to mutation in a gene on the mitochondrial circular chromosome and those due to mutation in a nuclear gene (autosome) affecting mitochondrial function.^11^ Typically, disorders due to mitochondrial gene mutation exhibit maternal transmission (i.e., transmitted from mothers to sons who do not transmit the disorder and to daughters who may transmit the disorder). The phenomenon of mitochondrial heteroplasmy, namely a variable proportion of normal and mutation‐containing mitochondria in each tissue, determines a certain phenotypic variability.

### 3.1 Mitochondrial encephalomyopathy with ragged‐red fibers (MERRF)

Although MERRF is phenotypically and genotypically heterogeneous, refractory myoclonic epilepsy is the clinical feature that distinguishes MERRF from other categories of mitochondrial disorders. Myoclonus is accompanied by generalized seizures, ataxia, migraine, muscular diseases, and neurocognitive degeneration. Lipomatosis can be a prominent clinical feature in some individuals. Muscular involvement can vary from a frank myopathy to exercise intolerance and muscle weakness, cardiac involvement, and impairment of the respiratory muscles leading to lactic acidosis in two thirds of cases. Neuropathy can also be seen.

The course can progress at different paces depending on the mutation and age of onset: It is usually slowly progressive in affected adults; however, some juveniles can exhibit a rapidly progressive course with a fatal outcome. MERRF is primarily due to pathogenic variants in the *MTTK* gene (~90% of MERRF patients) (OMIM #545000).^36^

### 3.2 Mitochondrial DNA polymerase gamma (POLG)‐related syndromes

Recessive mutations in the mitochondrial *POLG* are associated with a variety of phenotypic gestalts: mitochondrial recessive ataxia syndrome (MIRAS); myoclonic epilepsy myopathy sensory ataxia (MEMSA); spinocerebellar ataxia with epilepsy (SCAE) (OMIM #607459); sensory ataxia with neuropathy, dysarthria, and ophthalmoplegia (SANDO) (OMIM #607459). Alpers‐Huttenlocher syndrome (AHS) (OMIM #203700), characterized by progressive neurodegeneration, refractory seizures, movement disorder, neuropathy, and hepatic failure, is the most commonly reported phenotype of POLG‐related disease in children.

Seizures are predominantly focal motor, and *epilepsia partialis continua* is common, as is nonconvulsive status epilepticus—with and without visual features. Indeed, POLG‐related epilepsy may be suggested by occipital symptom predominance and asymmetric occipital lobe epileptic discharges. These occipital lobe seizures may not be obvious, as seizures consist of colored lights, scotoma, or visual blurring.^36^

## 4 MYOCLONIC EPILEPSY ASSOCIATED WITH CNVS

### 4.1 Down syndrome

Individuals with Down syndrome over the age of 40 years and with full 21 trisomy (OMIM #190685) may develop a neurologic condition called late onset myoclonic epilepsy in Down syndrome (LOMEDS). Patients present with spatial–temporal and language deficits, diffuse EEG abnormalities during sleep, and cerebral atrophy at neuroimaging. After a few months, upper limb myoclonic seizures typically appear upon awakening. The myoclonic jerks are time‐locked to diffuse polyspikes on EEG, and seizures are controlled by ASMs. Afterward, photosensitivity develops; epileptic and non‐epileptic myoclonus become persistent; cerebellar signs, severe dementia, and global decline are evident.^37^

### 4.2 Pallister‐Killian syndrome

Pallister‐Killian syndrome (PKS) (OMIM #601803) is a rare genetic disorder caused by a mosaic tetrasomy of the short arm of chromosome 12. Children with Pallister‐Killian syndrome (PKS) may show early myoclonic seizures spontaneously or triggered by low frequency (1–6 Hz) intermittent photic stimulation.^38^

## 5 MYOCLONIC EPILEPSY ASSOCIATED WITH NON‐CODING VARIANTS

### 5.1 Familial adult myoclonic epilepsy (FAME)

**FIGURE 5:**
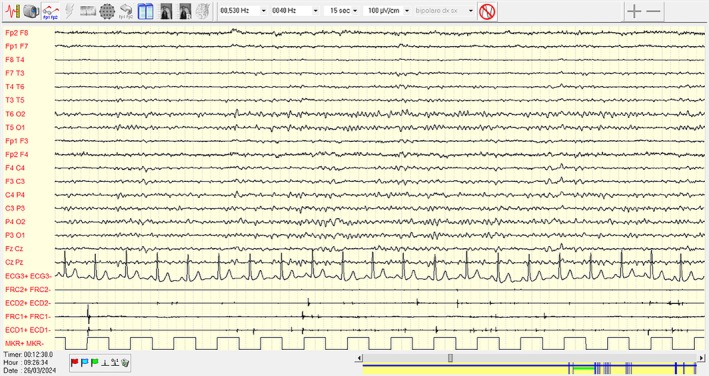
Polygraphic (EEG‐ECG‐EMG: FRC2 right flexor carpi radialis, ECD2 right common extensor digitorum, FRC1 left flexor carpi radialis, ECD1 left common extensor digitorum) recording of a patient with FAME2. On electroencephalogram, a normal background activity is seen. On electromyogram (EMG), frequent short, synchronous, and asynchronous arrhythmic bursts (<50 ms) of myoclonic activity without a simultaneous electroencephalographic discharge are recorded.

Familial adult myoclonic epilepsy (FAME) is an autosomal dominant, fully penetrant condition characterized by the occurrence of cortical tremor, myoclonus, and rare seizures. The onset is in the second decade of life, and the course is rather benign or slowly progressive. Indeed, epileptic seizures are easily controlled with ASMs that have both an antiseizure and an antimyoclonic effect, and individuals have a normal life expectancy. However, the myoclonus severity worsens with age, becoming disabling in the elderly.^49^, ^50^ Cortical tremor is the core feature of FAME (Figure 5) and is considered part of a spectrum of cortical myoclonus.^51^ Neurophysiological investigations, such as JL‐back AVG‐EEG and cortico‐muscular coherence analysis, giant somatosensory evoked potentials, and the presence of long loop reflex (or C‐reflex) at rest, support cortical tremor as the result of sensorimotor cortex hyperexcitability.^52^

Notwithstanding the obvious genetic etiology, its underlying molecular cause has remained elusive for decades. The history of FAME genetics, with the discovery of a new genetic mechanism unlinked to epilepsy before, represents the start of a new era in the genetics of epilepsy.

FAME has been described worldwide as geographic aggregates, especially in Japan and Europe, and initially linked to four main loci: 8q24 for the Japanese families,^53^ 2p11.1‐q12.2 for the Italian families,^54^ 5p for French and Dutch families,^54^ and 3q26.32‐3q28 for Thai families.^55^ A series of candidate gene variants were identified within these mapped intervals; however, none were replicated in other families with the same linkage. This unsuccessful finding led to the hypothesis that FAME could be caused by either non‐coding single nucleotide variants, structural variants, or repeat expansions. Genome sequencing data from an affected individual disclosed the insertion of a non‐coding TTTCA pentanucleotide repeat within a series of TTTTA repeats of *SAMD12* in *a* Japanese patient from an 8q24 linkage family (FAME1 OMIM #601068).^39^ This discovery led to the identification of a similar non‐coding pentanucleotide repeats, TTTCA and TTTTA, in all other families worldwide affecting different genes all lying within the linkage area discovered previously: *STARD7* for FAME 2 OMIM #607876, [52] *MARCH6* for FAME3 OMIM #613608,^41^ *YEATS2* for FAME4 OMIM #615127, [54] *TNRC6a* for FAME 6 OMIM #618074, and *RAPGEF2* for FAME7 OMIM #618075.^39^

More recently, TTTTA repeat expansions and TTTCA repeat insertions in intron 4 of the retinoic acid induced 1 (*RAI1*) gene were found in the first African family with FAME, from Mali.^43^

Regarding phenotype–genotype correlations, no major distinctions between the different genetic forms of FAME can be delineated. Anticipation, a common feature of most repeat expansion disorders which correlates with intergenerational increases in repeat lengths, seems to occur also in FAME families; however, this has only been studied at the molecular level for a few families and needs to be better elucidated.^2^

Interestingly, the genes encompassing the intronic TTTTA and inserted TTTCA repeat expansions have different molecular functions and do not all operate in the same biochemical pathways, although their protein products are expressed in the brain and mainly in the cerebellum. This suggests that the properties of the repeat and not the gene function are the important drivers of pathogenicity.^2^ The most accredited hypotheses consider toxicity due to the RNA molecules carrying UUUCA repeats, or toxicity of polypeptides encoded by the repeats, a mechanism known as repeat‐associated non‐AUG translation. These RNA or polypeptides would accumulate in the tissue and lead to neurodegeneration. This hypothesis is also corroborated by the analysis of postmortem brains of FAME1 expansion (in *SAMD12*) carriers that highlighted the presence of RNA foci.^56^

## 6 GENETIC ANALYSIS

Genetic testing should be initiated as soon as possible unless the course is benign, seizures represent the only symptom, and easily respond to treatment. They include different approaches that can be used sequentially: high resolution karyotype, array‐CGH, evaluation of candidate genes (individually or in gene panels), whole exome sequencing, mitochondrial gene sequencing, and whole genome sequencing.^11^ Contiguous gene syndromes suggested by the occurrence of multiple congenital abnormalities (such as a suspicion of Down syndrome or Pallister‐Killian) address the study toward a chromosome abnormality. These may be detected by karyotype (for large CNVs) or chromosome microarray analysis (for CNVs <1 MB).

Gene sequencing includes analysis of individual genes, panels of genes, and whole exome and whole genome sequencing. Multigene panels are preferred over single‐gene analysis because of cost effectiveness considering genetically heterogeneous syndromes. In addition, nowadays, most laboratories are equipped with whole exome sequencing technology and can sequence the whole exome directly. Thus, in case the analysis of multiple candidates' genes does not disclose a pathogenic variant, a new association can be looked up in the rest of the exome. On the contrary, whole genome sequencing is almost never available on clinical grounds and remains a research prerogative. This technology is today used to search for non‐coding variants. However, it represents the latest frontiers of genetic testing potentially able to detect all genetic abnormalities.

**FIGURE 6:**
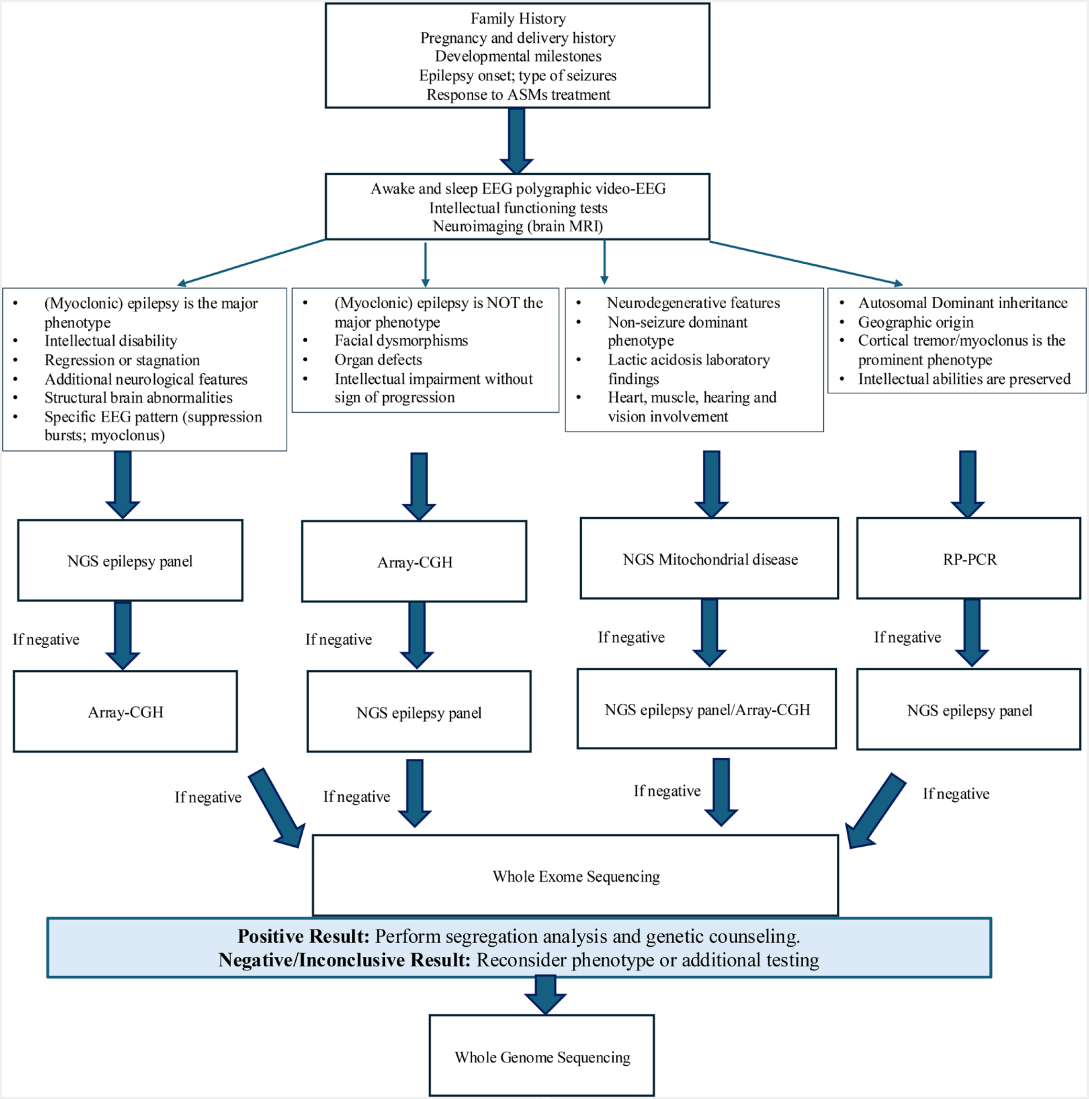
Diagnostic flowchart for patients with myoclonic seizures. CGH, comparative genomic hybridization; NGS, next generation sequencing; RP‐PCR, repeat‐primed polymerase chain reaction; WES, whole exome sequencing; WGS, whole genome sequencing.

When evaluating patients with ME associated with dysfunction in other organs such as the eye, muscle, and heart, it is worthy to test a panel of mitochondrial genes (i.e., mitochondrial genome sequencing, including mitochondrial gene deletion and panels of nuclear‐encoded mitochondrial proteins). Figure 6 shows a schematic flowchart to guide clinicians to investigate patients with myoclonic seizures.

### 6.1 Implication for counseling

Identifying the genetic cause of MEs is very important from different perspectives. First, it can often end a long diagnostic odyssey for families. Furthermore, having found the etiology provides the clinicians', patients', and families' necessary considerations for counseling, prognosis, comorbidities, and response to treatment.

It is important to say that while the segregation analysis is part of the genetic testing to confirm a clinical diagnosis, extending the analysis to siblings and other relatives is not always mandatory nor advisable. For instance, a younger sibling of a PME patient, who has not developed any symptoms and is not close to the age of disease onset, should not be tested until he or she reaches legal adulthood. Indeed, there is no advantage in knowing the genetic status beforehand, as only symptomatic treatments are available that would only be used when symptoms arise.

A proper familial counseling is mandatory in case of pregnancy if a progressive or severe condition has been confirmed genetically. The couple can benefit from pre‐implantation testing to plan a new pregnancy or be guided to fetal testing in case of an ongoing pregnancy. It goes without saying that these considerations are less imperative in the case of benign ME.

Ultimately, the goal of genetic testing is to delineate the syndrome as much as possible and eventually to provide a molecular target for precision medicine.

## 7 THERAPEUTIC IMPLICATIONS

Myoclonic seizures commonly worsen with the use of sodium channel blockers; however, there are exceptions. Indeed, sodium channel blockers are the treatment of choice in patients with mutations in *SCN8A*. Although the discussion about therapeutic implications goes beyond the scope of this review, it is of great value to consider that a molecular diagnosis could allow precision medicine treatment. Although this is not attainable for most of the cases yet, examples of enzyme replacement treatment and gene therapy are a reality for certain ME and are giving hope for future larger applications. Indeed, Cerliponase alfa is the available recombinant human tripeptidyl peptidase 1 (TPP1) enzyme replacement therapy for the treatment of CLN2, conferring a clinically meaningful slowing of the decline of motor and language function in children with CLN2 disease.^57^ With regard to Dravet Syndrome, an antisense oligonucleotide (STK‐001) aimed at upregulating NaV1.1 currents and restoring the function of GABAergic interneurons is currently tested in a human trial, and an adenoviral vector‐based gene therapy (ETX‐101) is scheduled for investigation.^58^

An antisense oligonucleotide (Gys1‐ASO) that targets the mRNA of the brain‐expressed glycogen synthase 1 gene (*GYS1*) has currently started the human trial for EPM2 after promising results in animal models. Indeed, the injection of Gys1‐ASO intracerebroventricularly has prevented Lafora body formation in young mice and inhibited further accumulation in older mice, markedly preventing large Lafora bodies characteristic of advanced disease.^59^

Although we believe that precision medicine represents the future for the treatment of these conditions, it is important to communicate to patients that the specific therapeutic implications in the case of a positive genetic diagnosis are currently few.

## 8 CONCLUSIONS

Myoclonic epilepsies often have a genetic basis, encompassing a spectrum of conditions with diverse clinical presentations and prognoses.

Myoclonic epilepsies can be associated with genetic heterogeneity, wherein the same clinical phenotype may result from mutations in different genes (e.g., in EEM+ and FAME). Similarly, myoclonic epilepsies can exhibit phenotypic variability, meaning that mutations in the same gene may lead to distinct syndromic phenotypes. For instance, mutations in the *SCN1A* gene can be responsible for both Dravet syndrome and EIDEE.

The rapid advancements in genetic research have significantly improved our understanding of their pathophysiology, revealing mechanisms ranging from single‐gene mutations to complex non‐coding repeat expansions. These discoveries have implications for precise diagnosis, prognosis, and genetic counseling. Despite these advances, many cases remain severe and unresponsive to conventional treatments. Precision medicine, though still in its infancy for most forms of MEs, offers a promising avenue for tailored therapies, as exemplified by emerging treatments such as enzyme replacement therapy and antisense oligonucleotides. Bridging the gap between genetic discoveries and clinical application remains a critical goal, with the hope that ongoing preclinical research will expand therapeutic options and improve outcomes for affected individuals. Continued investment in genetic technology and collaboration across research and clinical domains are essential to fully realize the potential of precision medicine in transforming the care of MEs patients. Through these efforts, we may move closer to providing effective, individualized treatments that address the unmet needs of this diverse patient population.

## AUTHOR CONTRIBUTIONS

A.C., conception and design of the study, collection of data, drafting, reviewing; A.R., M.R., images and table, reviewing, and final approval of the manuscript; P.S., conception and design of the study, revision, and final approval of the manuscript. All authors agree to be accountable for all aspects of the work.

## CONFLICT OF INTEREST STATEMENT

None of the authors has any conflict of interest to disclose. We confirm that we have read the Journal's position on issues involved in ethical publication and affirm that this report is consistent with those guidelines.

## ETHICS STATEMENT

All methods were performed in accordance with the ethical standards as laid down in the Declaration of Helsinki and its later amendments or comparable ethical standards.

## PATIENT CONSENT STATEMENT

Not applicable as no human subjects were involved in the study.

## Data Availability

The data that support the findings of this study are available in the article. If additional data were required, they might be requested from the corresponding author.
